# Apoptosis Induction, Cell Cycle Arrest and Anti-Cancer Potential of Tamoxifen-Curcumin Loaded Niosomes Against MCF-7 Cancer Cells

**DOI:** 10.30699/IJP.2022.124340.2356

**Published:** 2022-02-28

**Authors:** Mahdi Fatemizadeh, Farzaneh Tafvizi, Farzaneh Shamsi, Sahar Amiri, Afsaneh Farajzadeh, Iman Akbarzadeh

**Affiliations:** 1 Department of Neuroscience and Addiction Studies, School of Advanced Technologies in Medicine, Tehran University of Medical Sciences, Tehran, Iran; 2 Department of Biology, Parand Branch, Islamic Azad University, Parand, Iran; 3 Department of Microbiology, Faculty of Advanced Science and Technology, Tehran Medical Sciences, Islamic Azad University, Tehran, Iran; 4 Department of Genetics, Islamic Azad University, Tehran North Branch, Tehran, Iran; 5 Department of Chemical and Petrochemical Engineering, Sharif University of Technology, Tehran, Iran

**Keywords:** Breast cancer, Curcumin, Drug delivery, Niosome, Tamoxifen

## Abstract

**Background & Objective:**

Breast cancer is the most common cancer among women. One of the most effective treatments for breast cancer is chemotherapy, in which specific drugs destroy the mass and its proliferation is inhibited. Chemotherapy is the most effective adjunctive therapy when multiple medications are used concurrently. Also, combining the drugs with nanocarrier has become an important strategy in targeted therapy. This study is designed to assess the apoptosis induction, cell cycle arrest, and anti-cancer potential of Tamoxifen-Curcumin-loaded niosomes against MCF-7 Cancer Cells.

**Methods:**

A novel niosomal formulation of tamoxifen-curcumin with Span 80 and lipid to drug ratio of 20 was employed. The MCF-7 cells were cultured and then treated with IC_50_ value of tamoxifen-curcumin-loaded niosomes, the combination of tamoxifen and curcumin, tamoxifen, and curcumin alone. Flow cytometry, Real-Time PCR, and cell cycle analysis tests were conducted to evaluate the induction of apoptosis.

**Results:**

Drug-loaded niosomes caused up-regulation of *bax* and *p53* genes and down-regulation of *bcl2* gene. Flow cytometry studies showed that niosomes containing tamoxifen-curcumin increased apoptosis rate in MCF-7 cells compared to the combination of tamoxifen and curcumin owing to the synergistic effect between the two drugs along with higher cell uptake by formulation niosomal. These results were also confirmed by cell cycle analysis.

**Conclusion:**

Co-delivery of curcumin and tamoxifen using optimized niosomal formulation revealed that at acidic pH of MCF-7 cancer cells, released drugs from niosomal carriers would be more effective than physiological pH. This feature of niosomal nanoparticles can reduce the side effects of drugs in normal cells. Niosomal nanoparticles might be used as a biological anti-cancer factor in treatment of breast cancer.

## Introduction

According to WHO data, breast cancer affects one out of every eight to ten women. Breast cancer is the most common cancer in women and is caused by the proliferation of epithelial cells covering the breast ducts or lobes. It is also the second most common cause of cancer death and is a heterogeneous disease in clinical practice (1-3). Breast cancer treatment is easier in the early stages and may even be longer. The most common treatments currently used to treat cancer patients include chemotherapy and targeted therapy (4-8). One of the most effective methods is chemotherapy, in which specific drugs destroy the mass and its proliferation is inhibited. Chemotherapy may be used to treat a patient's recovery or prolong life and relieve pain. This treatment is not only limited to the drugs used to treat cancer but also includes antibiotics. Some researches have shown that chemotherapy is the most effective adjunctive therapy when multiple medications are used concurrently (5, 9). Curcumin has been shown to have anti-cancer properties in various cancer cell lines (10). Various mechanisms, including inhibition of aryl hydrocarbon receptor and cytochrome p450 1A1, p185 neu tyrosine kinase activity, KI67, PCNA, and P53 mRNA expression, have been suggested for the anti-cancer activity of curcumin in breast cancer cells (11). Curcumin can inhibit the proliferation of many tumor cell lines of different tissue origins. This effect varies depending on the cell type, curcumin concentration, and duration of treatment and is exerted by inhibition of the expression of cell cycle control genes in tumor cells (10, 12). Another drug widely used in the treatment of cancer is tamoxifen which is a non-steroidal estrogen receptor antagonist (11). According to the researches, anti-estrogenic drugs like tamoxifen can be helpful in treatmnent of breast cancer because estrogen plays an important role in its progression (13). In recent years, combining the drug with nanocarrier has become an essential strategy in targeted therapy. So that the drug may be covalently bonded to the nanoparticle surface or completely encapsulated in the drug carrier. The synthesis of nanocarrier drug molecules can protect drug degradation against enzymatic agents and provide the opportunity for targeted drug delivery and controlled release (14-17). Targeted drug delivery is more common in cancer treatment because the major challenge in cancer treatment is the deliberate elimination of cancer cells with the least side effects on healthy cells (18, 19). One type of nanocarriers is niosomes with a very small layered structure derived from a mixture of non-ionic surfactants of alkyl or diallyl polyglycerol ether and cholesterol and then hydration in the aqueous medium (20, 21). Niosomes, as vesicular delivery systems, are composed of non-ionic surfactants and cholesterol. Cholesterol enhances the rigidity of the bilayer (22-24). They can encapsulate both hydrophilic and lipophilic drugs in addition to acting as a drug carrier. (25). Niosomes have several advantages over liposomes, including higher stability, ease of storage for long periods, and lower cost (26, 27). Besides that, niosomes are biocompatible, non-immunogenic, biodegradable, structurally stable, and have increased antimicrobial activity and antibiotic therapeutic potency (28, 29).

The present study was a follow-up to the results of a previous study in which niosomal carriers containing both curcumin and tamoxifen were synthesized and optimized (19). Therefore, in this study, we evaluated the effects of curcumin and tamoxifen-free drugs and the niosomes containing these drugs on breast cancer cells to determine the mechanism of action of these compounds on cancer cells and hope for optimal treatment of this disease.

## Material and Methods

**Materials**

Tamoxifen (TMX) and Curcumin (Cur) were purchased from Iran Hormone and Exir Nano Sina companies, respectively (Iran). DMSO was obtained from Merck (Germany). Annexin V-FITC Flow cyto-metric kit was obtained from Affymetrix biosciences (USA). cDNA Synthesis kits (ER101-01) and RNA Extraction (AE301-02) were purchased from Transg-ene Biotech (China). MCF-7 cell line was taken from Pasteur Institute Cell Bank (Tehran, Iran). Phosphate-Buffered Saline (PBS) was from Merck Chemical Co. (Germany). PMI-1640, Formaldehyde, FBS, Trypsin-EDTA, Trypan blue, MTT, and Penicillin-/Streptomycin were obtained from Gibco (USA). 

**Preparation, Characterization, and Cytotoxicity Evaluation of TMX-Cur Loaded Niosomes**

Thin-layer hydration was used to synthesize and optimize the niosomal formulations containing TMX-Cur; this method's details are detailed in previous work (19). The size distribution of the synthesized form-ulation was investigated by nano Zetasizer. Encapsu-lation Efficiency of TMX**-**Cur loaded niosomes was determined by the ultrafiltration method presented in previous work. To investigate the cytotoxicity of the developed niosomes on MCF-7 cells, Cell Culture and MTT assay were used as described in previous work.

**Apoptotic Gene Expression Analysis**

In order to evaluate the expression of pro-apoptotic and anti-apoptotic genes, firstly, total RNA was extracted using an RNA extraction kit based on the manufacturer's protocols. The first-strand cDNA was synthesized using a cDNA Kit for a total volume of 20 μL. The Real-time PCR technique (SYBER Green method) was utilized to evaluate the expression of *bax, bcl2, and p53* genes in the treated cells with samples. The *actb* gene was used as an internal control. Each reaction mixture contained 12.5 µL of Master Mix (Bioneer, Korea); 1 µL of generated cDNA (0.1–1 µg of *actb* and intended genes); 1 µL (10 mM) of primers for each gene; and 9.5 µL of DEPC water. The primer sequences are shown in Table 1. Each gene's PCR reaction was done individually and three times. The real-time PCR conditions were tuned using the Bioneer Exicycler 96 system and the following schedule: a 10-minute initial denaturation phase at 95°C, followed by 40 cycles at 95°C for 20 seconds, 55°C for 40 seconds, and 72°C for 40 seconds. Rest software was used to calculate gene expression using the 2^−ΔΔCt^ technique. The primers of the target genes are given in Table 1. 

**Detection of Apoptosis by Annexin V-FITC**

MCF-7 cells (10^5^ cells/well) were treated with samples to determine the apoptosis/necrosis ratio (IC_50_ value) for 72 h, and then following with the manufacturer's instructions, the cells were analyzed using Annexin V/propidium iodide (PI) assay (Roche, Mannheim, Germany). MCF-7 cells that had not been treated served as a control. 

**Cell Cycle** **Analysis**

Cell proliferation was assessed using propidium iodide (PI) staining, and because DNA content is used to determine the cell cycle stage, PI binding to DNA is proportional to DNA content. At first, In 6-well plates, cells were seeded at a density of 1×10^6^ cells/well in complete medium and incubated overnight. After washing the cells with PBS, they were treated with drug patches for 72 h in a complete medium. Finally, the cells were fixed with ethanol 70% (18 h, 4°C), and were stained using 500 μL of PI solution (containing RNase) in the dark for 20 min at room temperature and then investigated by flow cytometry. All experiments were repeated three times. A flow cytometry device was used to perform the cellular analysis (Bio compare, USA). 

**Table 1:** Sequences of RT-PCR primers

Gene	Forward Primer	Reverse Primer
*BAX*	5’-CGGCAACTTCAACTGGGG-3’	5’-TCCAGCCCAACAGCCG-3’
*BCL-2*	5-’GGTGCCGGTTCAGGTACTCA-3’	5’-TTGTGGCCTTCTTTGAGTTCG-3’
*P53*	5’-CATCTACAAGCAGTCACAGCACAT-3’	5’-CAACCTCAGGCGGCTCATAG-3’
*ß-actin*	5-’TCCTCCTGAGCGCAAGTAC -3’	5’CCTGCTTGCTGATCCACATCT-3’

## Results

**Preparation, Characterization, and Cytotoxicity Evaluation of TMX-Cur Loaded Niosomes**

Based on the previous study (19), niosome formulation with Span 80 with lipid to drug ratio of 20 had optimal parameters, including size, PDI, and EE (size= 159.45 nm, PDI=0.192 and EE=98.37 and 96.40 for TMX and Cur). Also, the TMX-Cur loaded niosomes had a significant inhibitory effect against the MCF-7 cells compared to free TMX and Cur (19).

**Gene Expression Levels Analysis**

**Fig. 1:**
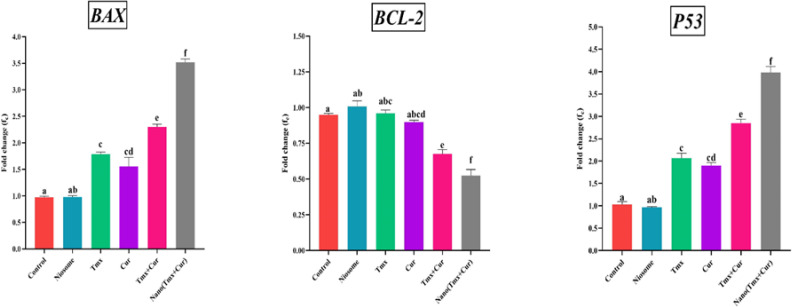
The expression of *bax*, *p53,* and *bcl-2* genes in MCF-7 cells after treatment with samples by real-time PCR method. There was no significant difference between the columns that have the same letter

TMX and Cur have been shown in studies to influence the expression of various genes in breast cancer cells. To see how niosomal formulation affects the treatment of breast cancer cells and the synergistic effect of TMX and Cur, expression of three different genes (*bax*, *bcl2*, and* p53*) was measured within the MCF-7 breast cancer cell line. It was demonstrated that the expression levels of *bax* and *p53 *genes were significantly increased (*P*<0.001) in TMX + Cur group compared with TMX, Cur, and control groups. The expression levels of nanoparticles containing TMX-Cur were significantly increased compared to the other treatments and control groups (*P*<0.001). The *bcl2* gene expression levels were downregulated in nanoparticles containing the TMX-Cur group compar-ed to the other treatments and control groups (*P*<0.001). Also, down-regulation of the* bcl2* gene for the TMX+Cur group in comparison with TMX, Cur, and control was significant (*P*<0.001) (Figure 1).

**Flow Cytometry**

The cancer cell was incubated with different samples at IC_50_ value for 72 h. In the flow cytometry, Annexin V is used as a sensitive probe with a high affinity to phospholipid phosphatidylserine, even when conjugated with FITC. In addition, PI can stain DNA in flow cytometry because of its intercalating property. The results showed that about 58.2%, 27%, 16.2%, and 10.37% of apoptosis was induced in MCF-7 cells after treatment with tamoxifen-curcumin loaded niosomes, a combination of tamoxifen and curcumin (TMX+Cur), tamoxifen, and curcumin alone, in comparison to the control group, which was significantly higher.

**Fig. 2:**
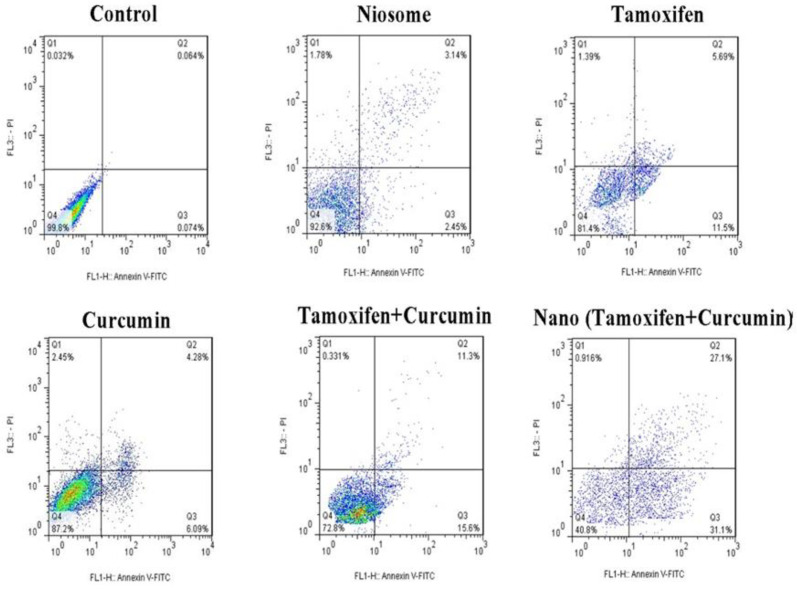
MCF-7 cell flow cytometry following treatment with several samples. The following information could be revealed by different quadrants in a scatter plot of double variable flow cytometry: Q1 (necrotic cells); Q2 (late apoptotic cells); Q3 (early apoptotic cells); Q4 (live cells)

**Fig. 3:**
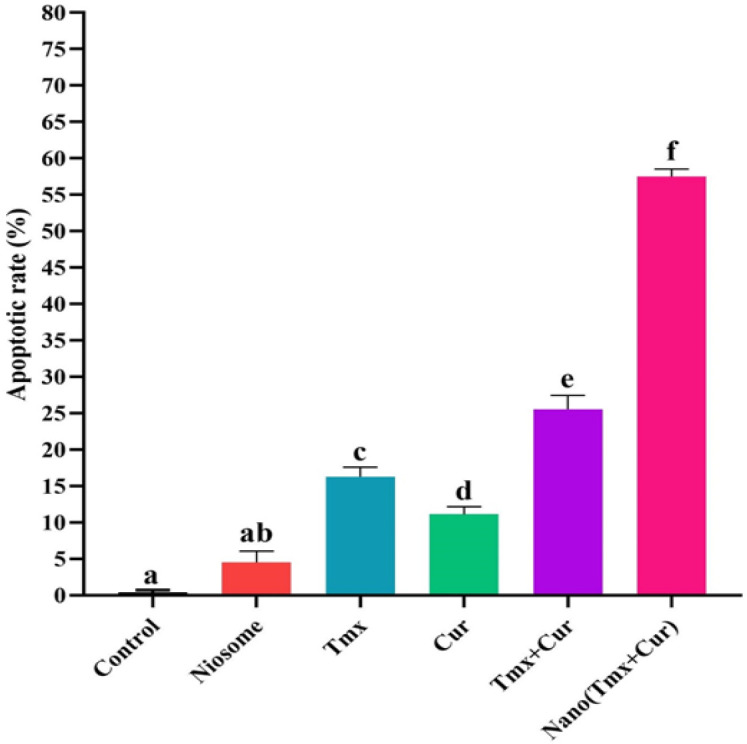
The quantitative apoptosis rate analysis of MCF-7 cells after treatment with samples by flow cytometry method. There was no significant difference between the columns that have the same letter

In Figures 2 and 3, quantitative apoptotic activity in MCF-7 cells by flow cytometry shows that the combination of TMX and Cur (TMX+Cur) significantly (*P*<0.001) increased the apoptosis of cancer cells compared to TMX, Cur, and control gro-ups. Also, niosomes containing two drugs significantly increased the apoptosis of cancer cells compared to the combination of the two free drugs and control groups (*P<*0.001).

**Cell Cycle**

**Fig. 4:**
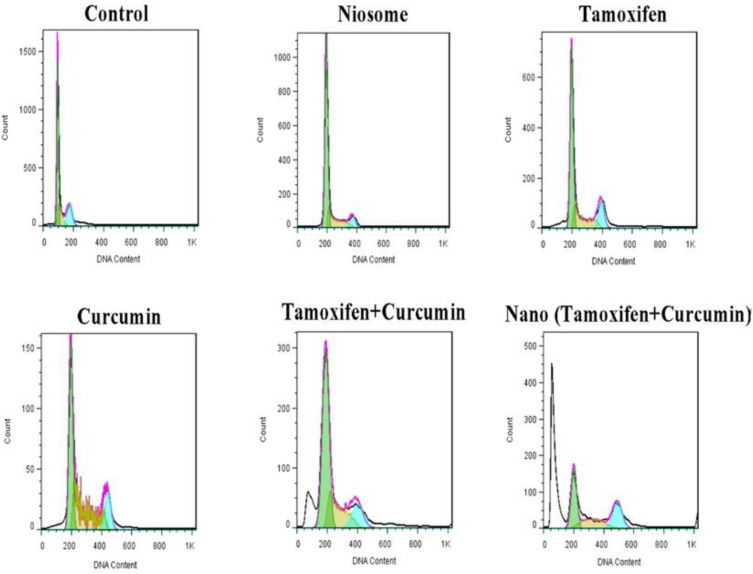
Cell cycle distribution for MCF-7 cells after treatment with different samples by flow cytometry method. The control sample refers to cells that have not been exposed to any drugs or nanomaterials

**Fig. 5:**
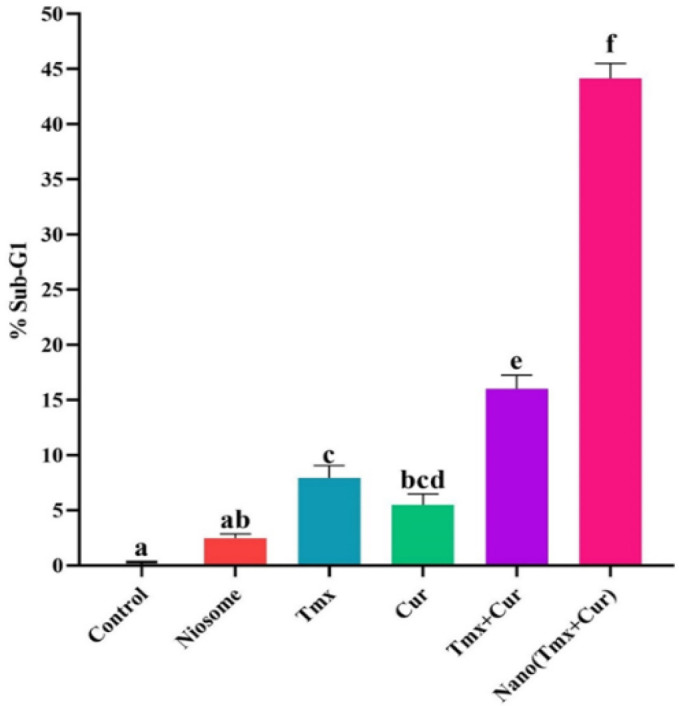
**Cell cycle analysis of different samples for MCF-7 cell line by flow cytometry** **method. There was no significant difference between the columns that have the same letter**

In this work, flow cytometry was used to examine their cell cycle (Figures 4 and 5). As shown in Figures 4 and 5**, **the synergistic effect of TMX-Cur loaded on the niosomes resulted in shifting towards a sub-G1 cell cycle stage in the breast cancer cell line (i.e., MCF- 7 cells: 16.01 % for TMX + Cur, and 44.16 % for TMX-Cur loaded niosome). These results confirmed an increase in apoptosis of the studied breast cancer cells.

## Discussion

Curcumin and tamoxifen are commonly used in the treatment of human lipid disorders. Many reports show anti-proliferative and anti-apoptotic activities against breast cancers. Nonetheless, such clinical applications are hindered due to the poor water-solubility of the drug (30, 31). Considering the properties of niosomes as efficient nanocarriers, in the current study, niosomal co-delivery of curcumin/tamoxifen to MCF-7 cancerous cells was investigated.

As the cell cycle test results showed, the proportion of cells in the Sub-G1 stage increased. The apoptotic cells were increased under the influence of niosomes containing both curcumin and tamoxifen and confirmed the inhibitory effect of niosomal carriers containing both drugs on cancer cell. It was also discovered that when two drugs were used together, the percentage of the cells in the Sub-G1 phase increased compared to each drug alone. Therefore, the combination of both drugs may have a more effective role in apoptosis of the cancer cells.

Tamoxifen and curcumin can be two notable drugs in treating breast cancer. In general, the increase in the cell population in the Sub-G1 phase following treatment with niosomes containing two drugs oftamoxifen, and curcumin, induced the highest apoptosis of the studied cancer cells (32, 33).

Genes can be classified into two groups: apoptosis and anti-apoptosis (34). On human chromosome 17's short arm, the *p53* gene is located, which is a type of tumor suppressor gene and can lead to alterations in important cellular functions, including apoptosis by encoding 53 KDa nuclear phosphoprotein (35, 36). The apoptosis rate in breast cancer cells can be facilitated by proper up-regulation of the above genes. Another apoptotic gene is *bcl2*, found in the mitochondrial outer and inner membranes. Low expression of the *bcl2* gene can activate caspase genes and lead to cell apoptosis (37, 38). It can be seen in Figure 5 that a combination of the two drugs, tamoxifen, and curcumin, work synergistically to increase the expression of the *bax* and *p53* genes while decreasing the expression of the *bcl2* gene in breast cancer. Encapsulation of tamoxifen and curcumin in niosomal nanocarriers also significantly enhances the synergistic effect of the two drugs because niosomal formulations increase the uptake of drugs by cancer cells. The transcriptional down-regulation and up-regulation of *bcl2* and *bax* genes confirmed these findings. According to research findings, the balance of pro-apoptotic and anti-apoptotic proteins, including the BAX and BCL2, is effective in cancer cell growth (6, 39, 40). In addition, it has been shown that P53 by increasing the expression of pro-apoptotic proteins such as BAX and BID regulates BAX: BCL2 rate. Because P53 interacts with BCL2, BAX and BID are activated and translocated in the mitochondrial outer membrane, and P53 can be transported directly to the mitochondria to activate the mitochondrial apoptotic pathway (41-43). 

## Conclusion

Co-delivery of TMX and Cur using optimized niosomal formulation with the smallest size, highest entrapment efficiency, and best stability evaluated in MCF-7 cell lines revealed that drugs released from niosomal carriers are controlled and sustained. This feature of niosomal nanoparticles can reduce the side effects of drugs in normal cells. Also, niosomal nanocarriers containing two drugs had a significant inhibitory effect compared to free drugs in the cancer cell line. Examining the expression of genes (i.e., *bcl2*, *bax*, and *p53*) also found that TMX-Cur loaded in the niosomes can up-regulate and down-regulate gene expression levels, leading to significant apoptosis in breast cancer cells. It is hoped that the results of this research will lead to a new and better way to help patients with cancer.

## Competing Interests

The authors declare there is no conflict of interest. 

## Ethics Statements

There were no "human subjects" in this study.

## Conflict of Interest

None.
